# Single-Locus and Multi-Locus Genome-Wide Association Studies for Intramuscular Fat in Duroc Pigs

**DOI:** 10.3389/fgene.2019.00619

**Published:** 2019-06-28

**Authors:** Rongrong Ding, Ming Yang, Jianping Quan, Shaoyun Li, Zhanwei Zhuang, Shenping Zhou, Enqin Zheng, Linjun Hong, Zicong Li, Gengyuan Cai, Wen Huang, Zhenfang Wu, Jie Yang

**Affiliations:** ^1^College of Animal Science and National Engineering Research Center for Breeding Swine Industry, South China Agricultural University, Guangdong, China; ^2^National Engineering Research Center for Breeding Swine Industry, Guangdong Wens Foodstuffs Group, Co., Ltd., Guangdong, China; ^3^Department of Animal Science, Michigan State University, East Lansing, MI, United States

**Keywords:** single-locus genome-wide association studies, multi-locus genome-wide association studies, candidate gene, intramuscular fat, Duroc pigs

## Abstract

Intramuscular fat (IMF) is an important quantitative trait of meat, which affects the associated sensory properties and nutritional value of pork. To gain a better understanding of the genetic determinants of IMF, we used a composite strategy, including single-locus and multi-locus association analyses to perform genome-wide association studies (GWAS) for IMF in 1,490 Duroc boars. We estimated the genomic heritability of IMF to be 0.23 ± 0.04. A total of 30 single nucleotide polymorphisms (SNPs) were found to be significantly associated with IMF. The single-locus mixed linear model (MLM) and multiple-locus methods multi-locus random-SNP-effect mixed linear model (mrMLM), fast multi-locus random-SNP-effect efficient mixed model association (FASTmrEMMA), and integrative sure independence screening expectation maximization Bayesian least absolute shrinkage and selection operator model (ISIS EM-BLASSO) analyses identified 5, 9, 8, and 21 significant SNPs, respectively. Interestingly, a novel quantitative trait locus (QTL) on SSC 7 was found to affect IMF. In addition, 10 candidate genes (*BDKRB2*, *GTF2IRD1*, *UTRN*, *TMEM138*, *DPYD*, *CASQ2*, *ZNF518B*, *S1PR1*, *GPC6*, and *GLI1*) were found to be associated with IMF based on their potential functional roles in IMF. GO analysis showed that most of the genes were involved in muscle and organ development. A significantly enriched KEGG pathway, the sphingolipid signaling pathway, was reported to be associated with fat deposition and obesity. Identification of novel variants and functional genes will advance our understanding of the genetic mechanisms of IMF and provide specific opportunities for marker-assisted or genomic selection in pigs. In general, such a composite single-locus and multi-locus strategy for GWAS may be useful for understanding the genetic architecture of economic traits in livestock.

## Introduction

In the past few decades, increasing lean meat content and reducing backfat thickness have been the main targets of pig breeding programs (Dong et al., 2014). However, due to insufficient attention to the quality of pork and negative correlation with selected traits, such as backfat thickness (Sellier et al., 2010), it has been challenging to satisfy consumer demand for meat quality (Hernandez-Sanchez et al., 2013). As a result, improvement of meat quality has become a priority (Fan et al., 2010; Franco et al., 2014). Intramuscular fat (IMF) is an important trait directly related to flavor and consumer acceptance (Sato et al., 2017; Won et al., 2018). Many studies have suggested that IMF influences pork tenderness, hydraulics, shearing force, and juicy flavor (Hocquette et al., 2010; Cho et al., 2015; Gong et al., 2018). In addition, muscles with sufficient IMF content are particularly suitable for conversion to dry products (Bosi and Russo, 2004).

Traditional methods for measuring IMF require chemical analysis [chemical methods to predict IMF (CIMF)] (Ramayo-Caldas et al., 2012; Pena et al., 2016; Sato et al., 2017) or spectral analysis (Prieto et al., 2009) after slaughter. The complexity of phenotyping makes it difficult to scale and greatly increases the difficulty and cost of genetic improvement. With the continuous development of color image processing technology, the reliability of using ultrasound images to predict IMF [ultrasonic methods to predict IMF (UIMF)] has become increasingly high (Newcom et al., 2002; Schwab et al., 2009). Jung et al. (2015) found that the genetic and phenotypic correlations for CIMF and UIMF were 0.75 and 0.76, respectively. Due to the low cost, convenience, high accuracy, and non-invasiveness of this technology (Cross and Belk, 1994; Yang et al., 2006; Jung et al., 2015), UIMF has been increasingly adopted in large-scale measurement of IMF.

The heritability of IMF in the literature has a relatively large range from 0.21 (Davoli et al., 2016) to 0.86 (Ciobanu et al., 2011), with an approximate average of 0.5 (Davoli et al., 2016), indicating a substantial genetic basis for this trait in pigs. However, the genetic mechanisms of IMF content are not clear. There are several biochemical and metabolic processes influencing fat deposition in muscle. These processes are determined by a set of interrelated genes and their interactions with environmental factors, including nutrition (Bolormaa et al., 2011; Moloney et al., 2013). At present, many studies have shown that IMF content of different breeds varies considerably (Garcia et al., 1986; Davoli et al., 2016). For instance, Chinese indigenous breeds are distinctively high in IMF content compared to main commercial breeds, among which the Duroc breed is the highest (Casellas et al., 2013; Meadus et al., 2018).

To date, there are 26,076 quantitative trait loci (QTLs) associated with 647 different pig traits mapped by previous studies (http://www.animalgenome.org/cgi-bin/QTLdb/SS/index). Among them, 213 QTLs have been identified for IMF traits, and most of these QTLs were identified using linkage mapping. However, directly using these QTLs for genetic improvement in pigs remains difficult due to the poor resolution of mapping as these loci are located within large intervals of at least 20 centimorgans (cM) in length (Tabor et al., 2002). As the cost of high-throughput commercial genotyping continues to decrease, genome-wide association studies (GWAS) have become one of the essential technology choices for genetic dissection of complex traits. The most common method is single marker analysis, testing one single nucleotide polymorphism (SNP) at a time and accounting for relatedness among the sample using either principal components as fixed covariates or a random polygenic effect using mixed linear model (MLM). Single-marker GWAS model has been successfully used to detect genetic markers for complex quantitative traits, including IMF (Jiao et al., 2014b; Ros-Freixedes et al., 2016; Duarte et al., 2018; Wang et al., 2019; Zhang et al., 2019). Most of these studies used CIMF, and the sample sizes were in the hundreds. Moreover, single-marker GWAS ignores the presence of multiple QTLs and may lead to severe bias in the point estimates of QTL effects and elevated type I and type II errors. Recently, several multi-locus GWAS methods have been developed to explicitly model multiple QTLs by treating them as random effects, which may increase the power to detect QTLs.

In the present study, we applied the single-locus analysis and three multi-locus methods, including the multi-locus random-SNP-effect mixed linear model (mrMLM) (Wang et al., 2016), the fast multi-locus random-SNP-effect efficient mixed model association (FASTmrEMMA) (Wen et al., 2017), and the integrative sure independence screening expectation maximization Bayesian least absolute shrinkage and selection operator model (ISIS EM-BLASSO) (Tamba et al., 2017), to decipher the genetic architecture of IMF in a large American Duroc population. The objective of this study was to use different methods to perform a comprehensive GWAS of IMF and delineate the genetic architecture.

## Materials and Methods

### Ethics Statement

The experimental procedures used in this study met the guidelines of the Animal Care and Use Committee of the South China Agricultural University (SCAU) (Guangzhou, People’s Republic of China). The Animal Care and Use Committee of the SCAU approved all animal experiments described in this study.

### Experimental Animals and Phenotyping

From 2013 to 2016, a total of 1,490 Duroc boars were collected from the Guangdong Wen’s Foodstuffs Group Co., Ltd. (Guangdong, China). All 1,490 boars were group housed in half-open cement-floor pens (10 animals in each pen, with an average of 2 m^2^ per pig) and fed under uniform feeding conditions for measurements of IMF during the fattening period (approximately 11 weeks) from 30 to 100 kg live weight. They were scanned with an Aloka 500V SSD ultrasound machine (Corometrics Medical Systems, USA) to measure IMF content in the *longissimus dorsi* muscle at the end of the test as previously described (Wang et al., 2017). The images were collected 6 to 7 cm off the midline across the tenth to eleventh ribs, and these images were used to predict IMF content with the BioSoft Toolbox for Swine software (http://www.biotronics-inc.com/Lesson%20Series%20One%20-%20Marbling.pdf).

### Genotyping

Genotyping was performed as described by Ding et al. (2018). Genomic DNA was extracted from ear tissue samples, and DNA quality was assessed by the ratios of light absorption (A_260/280_ and A_260/230_) and electrophoresis. All animals were genotyped with the Porcine SNP50 Beadchip from Illumina (San Diego, CA), which contains 50,703 SNP markers across the entire genome. Quality control was conducted using the PLINK tool (Purcell et al., 2007). Briefly, individuals with call rates > 0.95 and markers with call rates > 0.99, minor allele frequency > 0.01, and Hardy-Weinberg *P* value > 10^-6^ were retained. Moreover, all markers located on the sex chromosomes and unmapped regions were excluded from the analysis. A final set of 38,753 informative SNPs from 1,490 pigs were used for subsequent analyses.

### Single-Locus GWAS

The MLM was performed by using GEMMA (Zhou and Stephens, 2012; Zhou and Stephens, 2014). The statistical model is described as follows:

y=Wα+Xβ+u+ε;

$$u\sim {\text{MVN}}_{n}(0,\lambda \tau^{-1}K),\;\varepsilon \sim {\text{MVN}}_{n}(0,\;\tau^{-1}I_{n})$$

where *y* is the vector of phenotypic values for all pigs; *W* is the incidence matrix of fixed effects including live weight at the end of the test, and pig pen and year-season effects; α is the vector of corresponding coefficients including the intercept; *X* is the vector of SNP genotypes, and *β* is the corresponding effect of the marker; *u* is the vector of random effects, and ε is the vector of random residuals, both *u* and ε following the multivariate normal distribution; τ^−1^ is the variance of the residual errors; λ is the ratio between the two variance components; *K* is a standardized relatedness matrix estimated by the GEMMA software, which is the same as Yang et al. (2010); and *I*_n_ is an n × n identity matrix, and n is the number of animals. The relatedness matrix is slightly different from VanRaden (2008) in that the standardization was done at the SNP level.

Genome-wide significance was determined using the Bonferroni method by dividing the desired type I error level by the number of SNPs tested (Yang et al., 2005). The genome-wide significant thresholds were *P* < 0.05/N, where N is the number of SNPs. To cope with the false negative results of the Bonferroni correction being too conservative, we also set a more lenient threshold by multiplying the Bonferroni threshold by a constant of 20 (Yang et al., 2005).

The phenotypic variation of the genome’s heritability and significant SNP interpretation contributions was estimated by GCTA software (Yang et al., 2010; Yang et al., 2011).

### Multi-Locus GWAS Analysis

Three multi-locus GWAS approaches were employed using the R package “mrMLM” (Wang et al., 2016). All multi-locus approaches are divided into two stages. In the first step, SNP effects were treated as random; a small number of SNPs were selected based on the prior premise that most SNPs should have no effect on the quantitative traits. In the second step, all selected SNPs in the first step were placed into one multi-locus model. All three multi-locus methods require a centered response variable, that is, the phenotypic values need to be adjusted for fixed effects including principal components of the genotype matrix, live weight at the end of the test, and pig pen and year-season effects. Among the three multi-locus GWAS approaches, all parameters were set at default values except for the critical *P* value in the first step. In the first step, the critical *P* values were set at 0.001, 0.005, and 0.001 for mrMLM, FASTmrEMMA, and ISIS EM-BLASSO, respectively (Misra et al., 2017; Quan et al., 2018; Sant’Ana et al., 2018). It is worth mentioning that the critical LOD scores of all models are set to 3.0 in the second step.

### Haplotype Block Analysis

The haplotype block analysis is implemented by two softwares, PLINK (Purcell et al., 2007) and Haploview (Barrett et al., 2005) Linkage disequilibrium blocks were defined using Haploview based on SNPs with MAF values > 0.05, Mendelian errors < 2, and *P* values in the HWE test < 10^-3^ according to the criteria of Gabriel et al. (2002).

### Annotation of Candidate Genes and Pathway Enrichment Analysis

Search for potential candidate genes is based on the physical location of the significant trait-associated SNP locus in the recent *Sus scrofa* 11.1 genome [http://ensembl.org/Sus_scrofa/Info/Index]. Gene Ontology (GO) and Kyoto Encyclopedia of Genes and Genomes (KEGG) enrichment analysis on the identified candidate genes was further carried out using KOBAS 3.0 as described by Xie et al. (2011).

## Results

### Phenotype and SNP Data Statistics

The statistical distribution and heritability of IMF are shown in **Table 1**. The coefficient of variation (CV) of IMF is 11.42%, indicating that substantial phenotypic variation existed. The estimate of the genomic heritability of IMF was moderate (0.23 ± 0.04) (**Table 1**), indicating a genetic basis for IMF in Duroc pigs. The SNP distribution of the Porcine SNP50 Beadchip after quality control is provided in **S1 Table**. The average physical distance between two adjacent SNPs on the same chromosome was approximately 67.41 kb and ranged from 58.48 kb (SSC10) to 97.56 kb (SSC1).

**Table 1:** Phenotype and heritability statistics for IMF in Duroc pigs.

Trait	N	Mean (SD)	Min	Max	C.V.	h^2^(SD)
IMF	1490	2.54 ± 0.29	1.6	4.5	11.42	0.23 ± 0.04

Mean (standard deviation), minimum (Min), maximum (Max), coefficient of variation (C.V.), and heritability (standard deviation) of IMF values.

### SNPs Detected by Single-Locus GWAS

**Figure 1:**
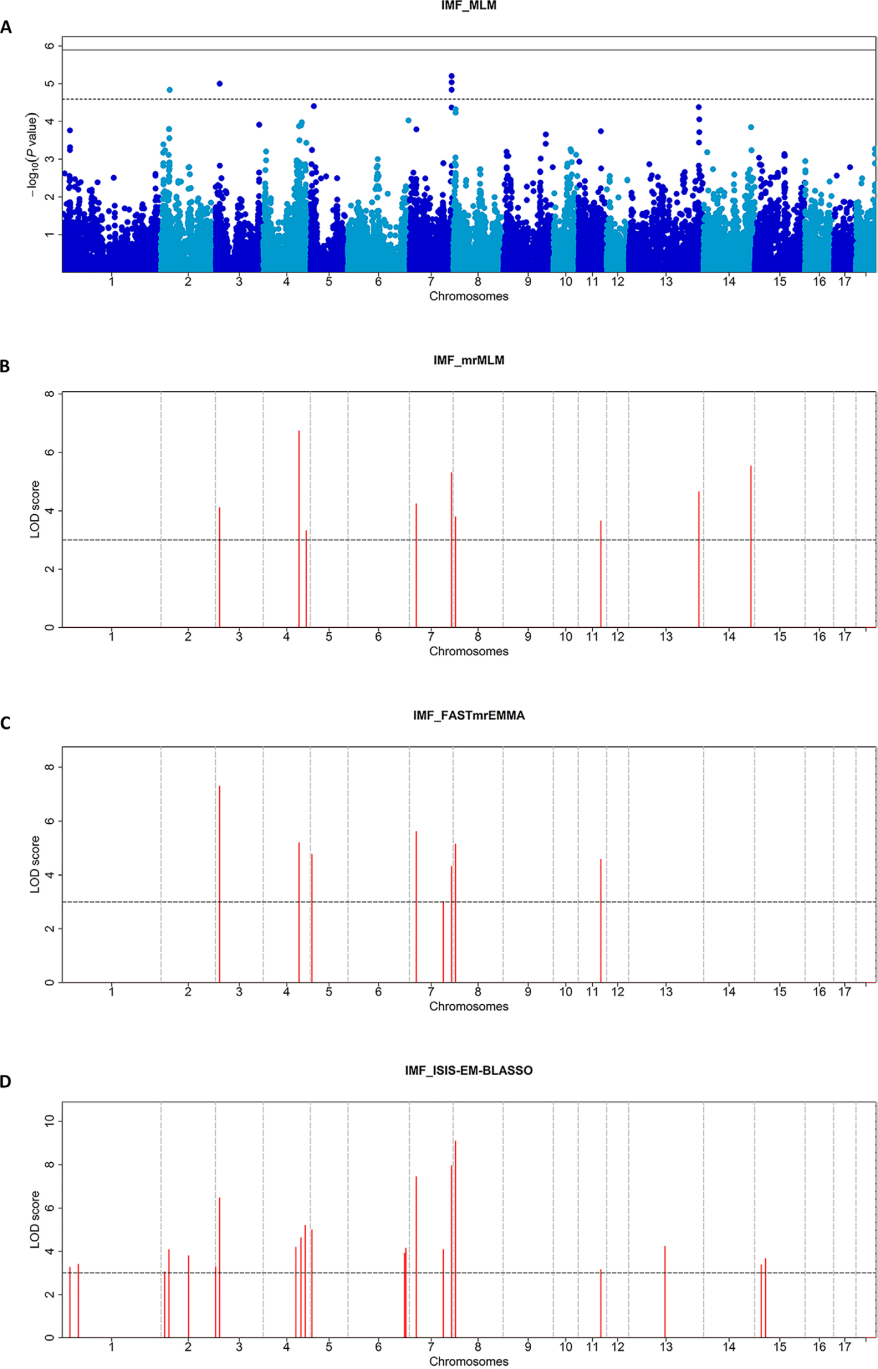
Manhattan plots of the MLM **(A)**, mrMLM **(B)**, FASTmrEMMA **(C)**, and ISIS EM-BLASSO **(D)** analyses for the IMF Trait in Duroc pigs. **(A)** The Manhattan plots indicate -log10 (*P* values) for genome-wide SNPs (y-axis) plotted against their respective positions on each chromosome (x-axis), and the horizontal line indicates the thresholds for significant (1.29E-06) and suggestive (2.58E-05) SNPs. **(B–D)** The Manhattan plots indicate LOD scores for genome-wide SNPs (y-axis) plotted against their respective positions on each chromosome (x-axis), and the horizontal lines indicate the thresholds for significance (LOD score = 3).

**Figure 2:**
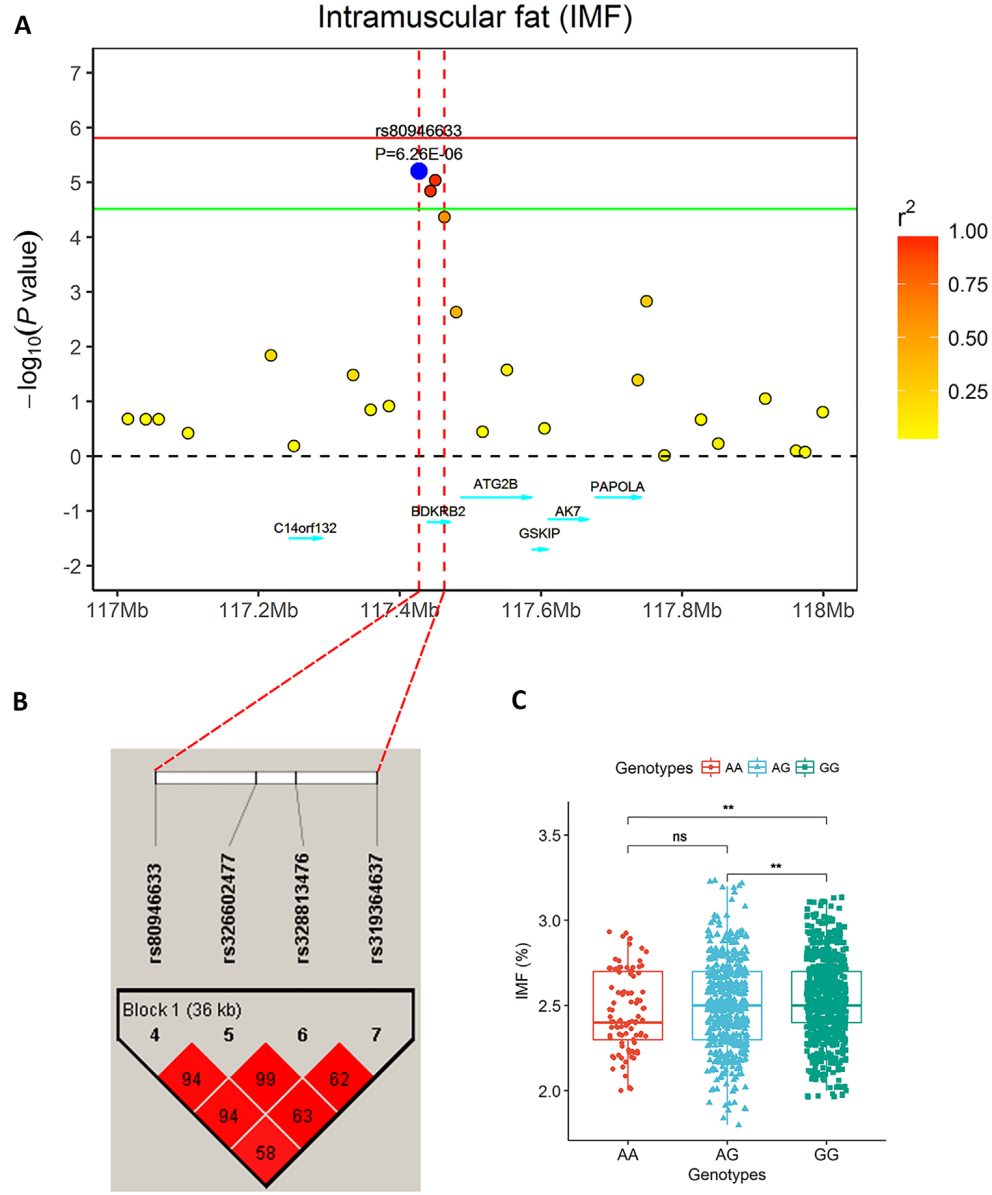
The significantly associated region for IMF on SSC7. **(A)** Regional plot of a 1 Mb region around the most significant SNP (rs80946633). The circular points represent the association significance measured by -log10 (*P*-values) that are plotted against genomic positions on the x-axis. Different colors indicate different linkage disequilibrium (LD) values between the top single nucleotide polymorphism (SNP) and other SNPs. **(B)** Haplotype blocks for significant SNPs indicate a haplotype block composed of significant SNPs located on SSC7 for the IMF trait. A haplotype block of 36 kb harbors the top SNP indicated in red and is highlighted by a black triangle. **(C)** The genotype effect plot of SNP rs80946633 (**P < 0.01, ns *P* > 0.05), which indicates SNPs with GG genotypes had higher IMF phenotypic values than those with genotypes AG and AA.

The single-locus GWAS result for IMF including their positions in the genome, their nearest known genes and distances, their *P* values, and genomic inflation factors (λ) are shown in **Table 2**, **Figure 1A**, and **Figure S1**. No significant SNPs were detected through a stringent genome-wide Bonferroni threshold (*P* < 1.29E−06). At a more lenient threshold (*P* < 2.58E−05) for suggestive associations, five SNPs were identified, and the proportion of phenotypic variance explained (PVE) by each SNP ranged from 1.45% to 2.08%. Two of them (rs328813476 and rs326602477) are located within the gene *BDKRB2* (bradykinin receptor B2), and another SNP (rs80946633) is 11.1 kb away from this gene. These three SNPs were mapped to one haplotype block spanning 36 kb affecting IMF on SSC7 (**Figure 2B**), among which the most significant SNP (rs80946633) explained 1.66% of the IMF phenotypic variance. For the lead SNP rs80946633, pigs with the GG genotypes had higher IMF phenotypic values than those with genotypes AG and AA (**Figure 2C** and **Table 3**). Of the remaining two SNPs, one (rs329147631) is 9.4 kb away from the *GTF2IRD1* (GTF2I repeat domain containing 1) gene, and the other SNP (rs341977270) is located approximately 0.4 Mb away from the *C11orf74* (chromosome 11 open reading frame 74) gene. In addition, QQ plots indicated that population stratification has been appropriately accounted for (Pearson and Manolio, 2008; Utsunomiya et al., 2013) (**Figure S1**).

**Table 2:** Description of significant SNPs identified by MLM as associated with IMF.

Marker	SSC^1^	Location (bp)^2^	*P* value^3^	r^2^ (%)^4^	Nearest gene	Distance^5^
rs341977270	2	24,096,039	1.46E-05	1.45	*C11orf74*	+379,675
rs329147631	3	11,528,693	1.00E-05	2.08	*GTF2IRD1*	+9,430
rs80946633	7	117,427,087	6.26E-06	1.66	*BDKRB2*	+11,134
rs326602477	7	117,443,751	1.45E-05	1.56	*BDKRB2*	within
rs328813476	7	117,450,278	9.15E-06	1.76	*BDKRB2*	within

^1^Sus scrofa chromosome.

^2^SNP positions in Ensembl.

^3^SNPs with P value come from SL-GWAS.

^4^r^2^ (%), phenotypic variation of traits explained by each marker.

^5^+/−: The SNP located in the upstream/downstream of the nearest gene.

**Table 3:** IMF (%) of different genotypes for SNP rs80946633.

Genotypes	N	Mean (SEM)^1^
AA	92	2.460 ± 0.024^a^
AG	562	2.497 ± 0.010^a^
GG	771	2.542 ± 0.009^b^

^1^Different letters indicate significant difference at P < 0.01.

### SNPs Detected by Multi-Locus GWAS

A total of 28 significant SNPs (LOD score > 3) were detected (**Table 4**, **Figure 1B–D**) by multi-locus methods. The mrMLM, FASTmrEMMA, and ISIS EM-BLASSO methods detected 9, 8, and 21 SNPs, respectively. Among all 28 significant SNPs, 7 SNPs were identified by at least two multi-locus GWAS methods, of which 3 SNPs (rs329147631, rs81397446, and rs341455185) were detected by all three multi-locus GWAS methods. Three SNPs (rs329147631, rs80946633, and rs328813476) were detected in both the single-locus and multi-locus models. Interestingly, the SNP rs329147631 located on SSC 3 was detected by all four methods. The other two SNPs (rs80946633 and rs328813476) are located in the haplotype block on SSC 7 that contains one gene, *BDKRB2* (**Figure 2B**).This was likely because the multi-locus GWAS methods properly accounted for linkage disequilibrium in this haplotype block, thus reducing the number of SNPs associated with the trait in this haplotype block (**Figure 2A**).

**Table 4:** Description of significant SNPs identified by multi-locus methods as associated with IMF.

Marker^1^	SSC^2^	Location (bp)^3^	LOD score	r^2^ (%)^4^	Nearest gene	Distance^5^	Method^6^
rs326526959	1	21,060,443	3.25	0.81	*UTRN*	−16,801	III
rs80909355	1	44,450,710	3.40	1.05	*ROS1*	within	III
rs81361093	2	10,135,131	3.79	0.12	*TMEM138*	within	III
rs81295735	2	22,147,009	4.07	1.64	*LRRC4C*	−513,328	III
NA	2	76,416,246	3.05	0.62	*AMH*	within	III
rs330719436	3	537,968	3.26	0.32	*GET4*	within	III
rs329147631	3	11,528,693	4.10, 7.30, 6.46	3.61, 1.81, 5.67	*GTF2IRD1*	+9,430	I, II, III
rs319425989	4	90,915,904	5.19	1.94	*APCS*	−13,984	III
rs80910035	4	99,522,059	6.73, 5.19	3.94, 1.13	*RNF115*	within	I, II
rs80785809	4	104,947,409	4.62	0.80	*CASQ2*	within	III
rs80782376	4	116,905,321	4.18	1.3	*S1PR1*	+158,750	III
rs339006733	4	119,633,516	3.31	2.14	*DPYD*	+297,877	I
rs81382770	5	4,713,576	4.77, 4.99	0.83, 1.80	*KIAA1644*	within	II, III
rs332805793	6	157,718,589	4.14	2.08	*MROH7*	−3,329	III
rs344352083	6	161,336,274	3.91	0.99	*FAF1*	within	III
rs81397446	7	19,581,991	4.23, 5.60, 7.45	1.24, 1.09, 1.88	*GMNN*	+3,955	I, II, III
rs320233990	7	94,642,773	3.01, 4.08	0.49, 1.83	*PCNX1*	−24,848	II, III
rs80946633	7	117,427,087	7.95	5.05	*BDKRB2*	+11,134	III
rs328813476	7	117,450,278	4.31	0.90	*BDKRB2*	within	II
rs319364637	7	117,463,409	5.30	3.71	*BDKRB2*	within	I
rs344928960	8	6,548,221	3.79, 5.14	1.11, 0.99	*ZNF518B*	+10,854	I, II
rs81319995	8	6,777,847	9.06	2.94	*CLNK*	−7,753	III
rs341455185	11	62,934,212	3.64, 4.57, 3.15	1.18, 0.75, 0.62	*GPC6*	+67,404	I, II, III
rs328211886	13	101,268,801	4.23	1.03	*OTOL1*	−74,382	III
rs321762476	13	195,215,153	4.65	2.09	*TIAM1*	within	I
rs80934705	14	131,949,286	5.52	2.82	*PLEKHA1*	+11,641	I
rs334840434	15	18,467,598	3.37	1.40	*MGAT5*	−548,039	III
rs81291577	15	30,615,152	3.65	0.85	*GLI2*	−36,070	III

^1^The name of the marker is obtained from NCBI, and NA indicates that the marker was not found in NCBI.

^2^Sus scrofa chromosome.

^3^SNP positions in Ensembl.

^4^r^2^ (%), phenotypic variation of traits explained by each marker.

^5^+/−: The SNP located in the upstream/downstream of the nearest gene.

^6^Method numbers correspond to (I) mrMLM, (II) FASTmrEMMA, and (III) ISIS EM-BLASSO.

### Candidate Genes and Functional Analysis

A total of 27 candidate genes that were located within or nearby the significant SNPs (*P* < 3.11E-05 or LOD score > 3) by all four methods were detected (**Tables 2** and **4**). Many candidate genes appear to have physiological roles that are relevant to IMF, including bradykinin receptor B2 (*BDKRB2*), GTF2I repeat domain containing 1 (*GTF2IRD1*), utrophin (*UTRN*), transmembrane protein 138 (*TMEM138*), dihydropyrimidine dehydrogenase (*DPYD*), calsequestrin 2 (*CASQ2*), zinc finger protein 518B (*ZNF518B*), sphingosine-1-phosphate receptor 1 (*S1PR1*), glypican 6 (*GPC6*), and GLI Family Zinc Finger 1 (*GLI1*). Interestingly, the subsequent gene enrichment analysis of 27 candidate genes for IMF found that several KEGG pathways and GO terms are significantly enriched for the candidate genes, including pathways related to the muscle contraction, muscle system process, developmental process, and sphingolipid signaling pathway, among others (**Table 5**).

**Table 5:** Significant KEGG PATHWAY and GO terms associated with IMF traits. (*P* < 0.05).

Term	Database	ID	Gene names	Corrected *P*-Value
Sphingolipid signaling pathway	KEGG PATHWAY	ssc04071	*BDKRB2, S1PR1*	9.89E-03
Muscle system process	Gene Ontology	GO:0003012	*BDKRB2, CASQ2, UTRN, TIAM1, GTF2IRD1*	4.69E-05
Growth	Gene Ontology	GO:0040007	*GLI2, TIAM1, PLEKHA1, S1PR1, ROS1, AMH*	0.000366
Organ morphogenesis	Gene Ontology	GO:0009887	*GLI2, TIAM1, PLEKHA1, S1PR1, GPC6, GMNN*	0.000395
Developmental process	Gene Ontology	GO:0032502	*APCS, UTRN, CASQ2, S1PR1, TMEM138, GTF2IRD1, PLEKHA1, ROS1, GLI2, GMNN, TIAM1, AMH, GPC6, LRRC4C*	0.000553
Regulation of developmental process	Gene Ontology	GO:0050793	*APCS, GPC6, GLI2, S1PR1, LRRC4C, TIAM1, AMH*	0.006229
Muscle contraction	Gene Ontology	GO:0006936	*BDKRB2, CASQ2, UTRN*	0.004588
Developmental growth	Gene Ontology	GO:0048589	*GLI2, PLEKHA1, S1PR1, TIAM1*	0.002635
Animal organ development	Gene Ontology	GO:0048513	*APCS, GPC6, TIAM1, GMNN, GLI2, UTRN, PLEKHA1, S1PR1, AMH*	0.002876

## Discussion

IMF content is an important meat quality trait that has received much attention. Among different breeds of pigs, many genome-wide association analyses have identified SNPs that are significantly associated with IMF (Schwab et al., 2009; Dong et al., 2014; Davoli et al., 2016; Ros-Freixedes et al., 2016; Wang et al., 2017; Won et al., 2018; Wang et al., 2019; Zhang et al., 2019). In the current study, as one of the largest GWAS for IMF, we identified 30 SNPs that were significantly associated with IMF by combining single-locus and multi-locus GWAS. The thresholds in previous GWAS varied widely, most were very lenient. Few SNPs would have been significant had more stringent threshold were used, regardless of whether the measurement method is CIMF (Hernandez-Sanchez et al., 2013; Davoli et al., 2016; Ros-Freixedes et al., 2016; Won et al., 2018; Wang et al., 2019) or UIMF (Jiao et al., 2014b; Wang et al., 2017). We did not replicate previous QTLs but identify a new QTL with relatively few SNPs associated with IMF by single marker analysis. Many factors can determine the specific SNPs and total number of SNPs associated with a trait. For instance, IMF is a complex quantitative trait with many genes each of small effects. In different studies, depending on the specific genetic backgrounds and sample size, different QTLs may be mapped. Moreover, phenotyping of animals is a challenge for this trait, and different studies may not be measuring exactly the same location of the muscle for IMF. This could contribute to the differences between studies. While we are not able to pinpoint specific reasons for the disagreement between studies, our study represents one of the largest GWAS to date for IMF. Interestingly, among the 30 significant SNPs, the PVE of eight of them is greater than 2% in at least one GWAS method, and the PVE of rs80946633 and rs329147631 was even greater than 5%. Although these molecular markers are not QTN for IMF, higher PVE implies that these markers can be used in molecular marker-assisted selection and genome selection in pigs to increase IMF content in pigs.

In the present study, we estimated the genomic heritability of the IMF to be 0.23, obtained by whole genome dense markers. This is similar to the result of Jiao et al. (2014a) (the genomic heritability is 0.27), in which the IMF is also measured by the UIMF method. In addition, many studies have found that the genomic heritability was generally lower than the heritability from single-trait animal models (Kim et al., 2012; Jiao et al., 2014a; de los Campos et al., 2015). This may be the result of “missing heritability” and is also a controversial issue in human genetics (Manolio et al., 2009; Eichler et al., 2010).

The IMF QTL on SSC7 discovered in this study has not been reported before for association with IMF. However, the QTL overlaps with previously identified QTLs associated with other meat quality traits. For example, Cho et al. (2015) found that this region was associated with loin fat percentage and muscle moisture percentage in a large F2 intercross between Landrace and Korean native pigs. Edwards et al. (2008) also found that this region was associated with 45 min–24 h pH decline in an F_2_ Duroc × Pietrain resource population.

### Potential Candidate Genes Reveal the Possible Molecular Basis of IMF

In the current study, we found SNPs significantly associated with IMF in a haplotype block spanning 36 kb on SSC 7, which contains only one known gene, *BDKRB2* (bradykinin receptor B2). *BDKRB2* is a protein coding gene that is normally highly expressed in muscles, such as smooth muscle (Marcadenti, 2015). Bradykinin is an inflammatory mediator with vasodilation activity and exerts its effects *via* two receptor subtypes (the B1 and B2 receptors) (Saunders et al., 2006). In adipose tissue, bradykinin may stimulate proinflammatory interleukins, such as IL-6 and IL-8, through its action on receptors expressed by adipocytes (Catalioto et al., 2013). However, the overexpression of the bradykinin receptor 2 gene in adipocytes can cause excessive secretion of proinflammatory cytokines and lead to endocrine disorders (Eder et al., 2009). Endocrine disorders can cause excessive expansion of adipose tissue, eventually increasing the risk of obesity (Hajer et al., 2008). In addition, in obese ob/ob mice, B2 receptor activity is also detected in the white adipose tissue, which, along with the hypothalamus, is the main site of neuroendocrine regulation of energy metabolism (Abe et al., 2007). This implies a close relationship between the kallikrein-kinin system and obesity. Therefore, the *BDKRB2* gene may be a strong candidate gene for IMF due to its influence on fat metabolism.

The rs329147631 SNP was detected by all four GWAS methods, and it is located near *GTF2IRD1*. This gene is most prominently expressed in human brown fat adipocytes and is expressed to a lesser extent in smooth muscle (Palmer et al., 2007). Mera (2014) found that *GTF2IRD1* is a PRDM16-interacting transcription factor that is enriched in beige and brown fat cells. In addition, *GTF2IRD1* is enriched in brown adipose tissue and is increased in beige and brown fat in response to beta-3-adrenergic stimulus (Mera, 2014). *GTF2IRD1* may be an important regulator of beige fat differentiation.

The rs326526959 SNP is close to the protein coding gene *UTRN* (utrophin). This gene shares both structural and functional similarities with the dystrophin gene. Myogenesis, adipogenesis, and chondrogenesis are impaired in adipose-derived stem cells from utrophin/dystrophin double-knockout mice. It was indicated that the *UTRN* plays an important role in the differentiation of adipose-derived stem cells into adipocytes (Sohn et al., 2013). This suggests that *UTRN* plays key role in the formation and growth of adipose tissue.

The rs81361093 SNP is located inside the *TMEM138* (transmembrane protein 138) gene, which encodes a multi-pass transmembrane protein. Although there are currently no reports of *TMEM138* involved in fat growth and development, the related genes *TMEM120A* and *TMEM120B* are highly expressed in fat, and *TMEM120A* and *TMEM120B* knockdown individually and together affect adipocyte differentiation and metabolism in mice (Batrakou et al., 2015). Another homologous gene, *TMEM26,* was identified as a cell surface marker of a natural beige adipocyte precursor. The differentiation of *CD137* ^+^ *TMEM26* ^+^ precursor cells to *UCP1* ^+^ beige adipocytes is regulated by β-adrenergic receptor agonists (Harms and Seale, 2013). In addition, another homologous gene, *TMEM60*, was associated with increased marbling fat in a study of candidate genes for the marbling traits of cattle, and the candidate gene *DPYD* (dihydropyrimidine dehydrogenase) also affected the marbling of beef (Lim et al., 2014). The significantly associated rs339006733SNP is closest to the *DPYD* gene. Therefore, *TMEM138* and *DPYD* could be potential candidate genes for IMF.

The rs80785809 SNP was detected by two GWAS methods (MLM and ISIS EM-BLASSO) and is located inside the *CASQ2* (calsequestrin 2) gene. This gene encodes a calcium-binding protein that stores calcium for muscle function. Clark et al. (2011) identified a specific bovine gene related to IMF deposition that is expressed in skeletal muscle and found that the *CASQ2* gene is highly expressed in muscles with high IMF content in beef cattle. In addition, Mills et al. (2011) found that loss of PRDM16 promotes the differentiation of brown fat precursors into skeletal muscle, and the *CASQ2* gene may be involved in its regulation.

*ZNF518B* (zinc finger protein 518B) belongs to the zinc finger protein family gene. Other zinc finger protein family genes, such as zinc finger protein 467 (*ZFP467*) and zinc finger protein 36 (*ZFP36*), were reported to be involved in the differentiation and regulation of adipocytes. *ZFP467* can regulate the differentiation of adipose-derived stem cells (You et al., 2015); *ZFP36* was identified as a candidate gene for obesity-related metabolic complications (Bouchard et al., 2007). Considering that zinc finger protein family genes are reported to be involved in multiple processes of fat development, differentiation, and deposition, we infer that it may be an important candidate gene that affects IMF, although there are no current studies on the role of *ZNF518B* in fat.

Sphingosine-1-phosphate receptor 1 (*S1PR1*) has been reported to be related to obesity (Nagahashi et al., 2018). In addition, this gene plays an important regulatory role in the proliferation and differentiation of adipose precursor cells, and blocking its homologue *S1PR2* induces proliferation and suppresses differentiation of (pre)adipocytes both *in vivo* and *in vitro* (Kitada et al., 2016).

The rs341455185 SNP was detected by three multi-locus GWAS methods, and its closest gene is *GPC6* (glypican 6). The glypicans comprise a family of glycosylphosphatidylinositol-anchored heparan sulfate proteoglycans, and they have been implicated in the control of cell growth and cell division. *GPC4* has been reported to interact with insulin receptors, enhance insulin receptor signaling, and enhance adipocyte differentiation (Ussar et al., 2012). In addition, *GPC4* may be involved in regulating obesity and body fat distribution (Liu et al., 2014). *GPC3* is a potential target gene for microRNA *Mir717*, and the genes that *Mir717* may target are related to mammalian obesity and other related phenotypes (Kunej et al., 2010).

During the fetal and neonatal stages, muscle cells and adipocytes (fat cells) are all derived from mesenchymal stem cells (MSCs). The majority of MSCs develop into myogenic cells, but a small portion of these cells differentiate into adipocytes, which are the basis for IMF accumulation that produce marbling in offspring (Du et al., 2010). *GLI2* is one of the three glioma-associated oncogenes (GLIs), including *GLI1*, GLI2, and *GLI3*. *GLI2* is a transcriptional regulator in the Hedgehog signaling pathway (Mahindroo et al., 2009). The Hedgehog signaling pathway has fundamental roles in the formation of tissue patterns during embryonic development, and sonic hedgehogs (SHH) is one of the hedgehog proteins in mammals. A growing body of evidence suggests a role for SHH signaling in adipogenesis. For instance, obesity downregulates SHH signaling, including the expression of *GLI1*, *GLI2,* and *GLI3* (Suh et al., 2006). However, activation of SHH signaling inhibits adipogenesis in 3T3-L1 and C3H10T1/2 cells (Zehentner et al., 2000; Spinella-Jaegle et al., 2001; Cousin et al., 2007). Therefore, *GLI2* could affect adipocyte differentiation and adipogenesis by regulating the Hedgehog signaling pathway and is a functionally plausible candidate gene for IMF content in pork.

Functional annotation revealed a number of pathways and biological processes that are significantly overrepresented among the 27 positional candidate genes for IMF (**Table 5**). Most of the significantly GO terms are related to muscle development processes, such as muscle system process and muscle contraction. Considering that the deposition of fat in muscle is closely related to the overall growth and development of the muscle, it is conceivable that these genes may be involved in various stages of growth of the muscles and thus affect the deposition of fat. Interestingly, the sphingolipid signaling pathway is associated with fat accumulation and was the only KEGG pathway detected as significantly enriched in this study. Choi and Snider (2015) found that overnutrition associated with a high-fat diet (HFD) increases sphingomyelin and sphingosine-1 phosphate (S1P) levels in adipose tissue through the sphingolipid metabolic pathway, leading to dysregulation of lipid accumulation and exacerbating obesity-related conditions. The accumulation of ceramide is a metabolic hub for sphingolipid metabolism, while ceramide accumulation is sufficient to induce an obesity phenotype and fat storage (Walls et al., 2013).

### Effectiveness of the Multi-Locus GWAS Approaches

The standard method used in GWAS is single-**locus** analysis, such as one that uses a mixed linear model (Yu et al., 2006). Despite its simplicity and speed, single-locus analysis makes a strong assumption that only one QTL has effect. This is largely valid for polygenic traits, where QTLs other than the one being tested can be properly accounted for by the polygenic term. In this study, we used both the single-locus analysis and multi-locus analysis to overcome some of the limitations in single-locus analysis. Multi-locus methods including mrMLM, FASTmrEMMA, and ISIS EM-BLASSO (Wang et al., 2016; Tamba et al., 2017; Wen et al., 2017) were applied. Standard multi-locus GWAS has two stages (Shi et al., 2011). In the first stage, a candidate subset of markers is selected through single-locus MLM. After this stage, putative markers are added to the model iteratively until a certain selection criterion is met (Wang et al., 2016). Such multi-locus model can reduce bias in the effect estimates and improve power to detect associations (Ma et al., 2018; Zhang et al., 2018). By combining both single-locus and multi-locus methods, we found 10 candidate genes that appeared to have IMF-related biochemical and physiological effects. Among the 10 strong candidate genes, the single-locus method found two (*BDKRB2* and *GTF2IRD1*), while the multi-locus method found all candidate genes. Interestingly, the two candidate genes (*BDKRB2* and *GTF2IRD1*) were detected by all multi-locus methods. This suggests that multi-locus methods are able to detect candidate genes that elude single-locus methods. In general, our study demonstrated that improved efficiency and accuracy could be achieved by a combination of the single-locus and multi-locus GWAS for identification of IMF-related QTLs in pigs.

## Conclusions

In this study, we used a combined strategy (including single-locus and multi-locus methods) to perform GWAS based on genomic data sets to identify new associations. We successfully identified a new genomic region and 10 new genes related to IMF. The GO analysis showed that most of the genes are involved in the muscle system process. The identification of the QTLs and candidate genes that are associated with IMF in the present study may contribute to marker-assisted selection in pig breeding.

## Data Availability Statement

All genotypic data were deposited in Figshare https://figshare.com/s/be3bb2047df324c8a77e.

## Author Contributions

JY, ZW, and WH conceived and designed the experiments. RD, MY, JQ, SL, ZZ, and SZ performed the experiments. RD, JY, and WH analyzed the data and wrote the article. MY, EZ, LH, ZL, and GC collected the samples and recorded the phenotypes. ZW contributed the materials. All authors reviewed and approved the article.

## Funding

This study was supported by the National Natural Science Foundation of China (grants 31601912 and 31790411), the Science and Technology Planning Project of Guangdong Province (grant no. 2017B020201012), the Pearl River Nova Program of Guangzhou (grant no. 201906010011), and the Natural Science Foundation of Guangdong Province (grants 2018B030315007 and 2017A030313213).

## Conflict of Interest Statement

Ming Yang and Zhenfang Wu who providing all phenotypic data and ear tissue were employed by Wens Foodstuffs Group, Co., Ltd. This does not alter our adherence to Frontiers in Genetics policies on sharing data and materials.

The remaining authors declare that the research was conducted in the absence of any commercial or financial relationships that could be construed as a potential conflict of interest.
