# Supplementary material for: Single-Locus and Multi-Locus Genome-Wide Association Studies for Intramuscular Fat in Duroc Pigs
Source: Front Genet. 2019 Jun 28;10:619. doi: 10.3389/fgene.2019.00619 (PMC6609572; doi:10.3389/fgene.2019.00619)
Supplement: Supplementary file 1 [file Table_1.docx]

**Supplementary Materials**

**Single-locus and multi-locus genome-wide association studies for intramuscular fat in Duroc pigs**

Rongrong Ding^1,$^, Ming Yang^2,$^, Jianping Quan^1^, Shaoyun Li^1^, Zhanwei Zhuang^1^, Shenping Zhou^1^, Enqin Zheng^1^, Linjun Hong^1^, Zicong Li^1^, Gengyuan Cai^1,2^, Wen Huang^3^, Zhenfang Wu^1,2,*^, Jie Yang^1,*^

^1^College of Animal Science and National Engineering Research Center for Breeding Swine Industry, South China Agricultural University, Guangdong 510642, P.R. China.

^2^National Engineering Research Center for Breeding Swine Industry, Guangdong Wens Foodstuffs Group, Co., Ltd., Guangdong, China.

^3^Department of Animal Science, Michigan State University, East Lansing, MI, United States

^$^ These authors have contributed equally to this work

**Correspondence and requests for materials should be addressed to J.Y. (email:* [*jieyang2012@hotmail.com*](mailto:jieyang2012@hotmail.com)*) , Z.W. (email: wzfemail@163.com*)

**S1 Table. Distribution of SNPs after QC and the average distance between adjacent SNPs on each chromosome.**

| **SSC** | **SNP no.** | **Physical size (Mb)^1^** | | **kb/SNP** |
| --- | --- | --- | --- | --- |
| 1 | 2812 | 274.33 | 97.56 | |
| 2 | 2179 | 151.94 | 69.73 | |
| 3 | 1966 | 132.85 | 67.57 | |
| 4 | 2053 | 130.91 | 63.77 | |
| 5 | 1632 | 104.53 | 64.05 | |
| 6 | 2232 | 170.84 | 76.54 | |
| 7 | 2026 | 121.84 | 60.14 | |
| 8 | 2029 | 138.97 | 68.49 | |
| 9 | 2078 | 139.51 | 67.14 | |
| 10 | 1186 | 69.36 | 58.48 | |
| 11 | 1348 | 79.17 | 58.73 | |
| 12 | 957 | 61.6 | 64.37 | |
| 13 | 2299 | 208.34 | 90.62 | |
| 14 | 2208 | 141.41 | 64.04 | |
| 15 | 1992 | 140.41 | 70.49 | |
| 16 | 1260 | 79.94 | 63.44 | |
| 17 | 999 | 63.49 | 63.55 | |
| 18 | 894 | 55.98 | 62.62 | |
| Total | 32446 | 2265.42 |  | |

SNP, single nucleotide polymorphisms; SSC, *Sus scrofa* chromosome

^1^The physical size is based on *Sus scrofa* Build 11.1
