# Supplementary material for: Single-Locus and Multi-Locus Genome-Wide Association Studies for Intramuscular Fat in Duroc Pigs
Source: Front Genet. 2019 Jun 28;10:619. doi: 10.3389/fgene.2019.00619 (PMC6609572; doi:10.3389/fgene.2019.00619)

Supplementary Materials

Single-locus and multi-locus genome-wide association studies for intramuscular fat in Duroc pigs

Rongrong Ding[1,$], Ming Yang[2,$], Jianping Quan[1], Shaoyun Li[1], Zhanwei Zhuang[1], Shenping Zhou[1], Enqin Zheng[1], Linjun Hong[1], Zicong Li[1], Gengyuan Cai[1,2], Wen Huang[3], Zhenfang Wu[1,2,*], Jie Yang[1,*]

[1]College of Animal Science and National Engineering Research Center for Breeding Swine Industry, South China Agricultural University, Guangdong 510642, P.R. China.

[2]National Engineering Research Center for Breeding Swine Industry, Guangdong Wens Foodstuffs Group, Co., Ltd., Guangdong, China.

[3]Department of Animal Science, Michigan State University, East Lansing, MI, United States

[$] These authors have contributed equally to this work

**Correspondence and requests for materials should be addressed to J.Y. (email: jieyang2012@hotmail.com) , Z.W. (email: wzfemail@163.com)*

S1 Fig. Quantile–quantile (Q–Q) plots of single-locus GWAS for IMF in Duroc pigs. Q-Q plots show the observed versus expected log *P*-values.

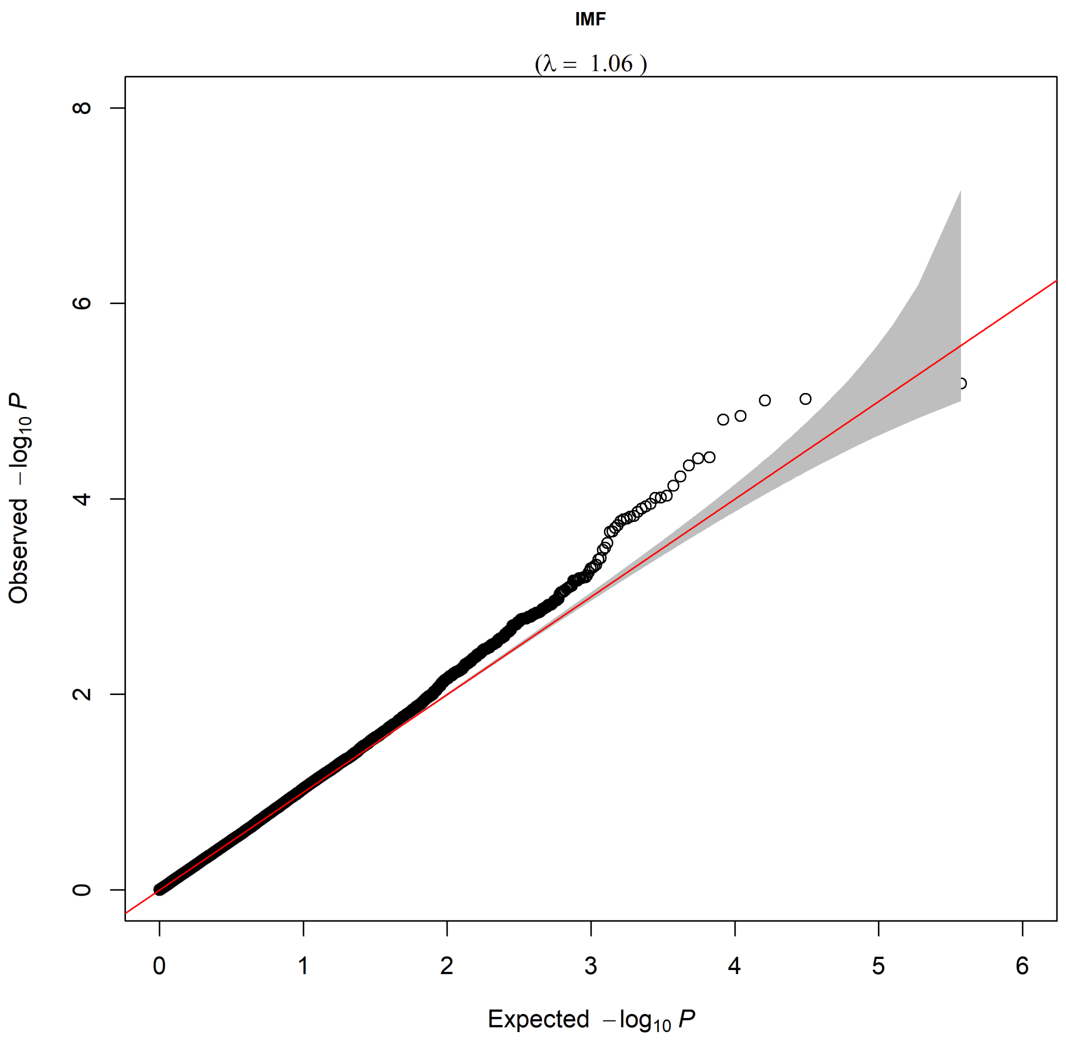

Supplement: Supplementary file 2 [file Image_1.pdf]
